# MicroRNA 34a–AXL Axis Regulates Vasculogenic Mimicry Formation in Breast Cancer Cells

**DOI:** 10.3390/genes12010009

**Published:** 2020-12-23

**Authors:** Dansaem Lim, Jin Gu Cho, Eunsik Yun, Aram Lee, Hong-Yeoul Ryu, Young Joo Lee, Sukjoon Yoon, Woochul Chang, Myeong-Sok Lee, Byung Su Kwon, Jongmin Kim

**Affiliations:** 1 Division of Biological Sciences, Sookmyung Women’s University, Seoul 04310, Korea; lds05101@hanmail.net (D.L.); jgcho84@gmail.com (J.G.C.); yes951212@naver.com (E.Y.); aram0918@sookmyung.ac.kr (A.L.); yoonsj@sookmyung.ac.kr (S.Y.); mslee@sookmyung.ac.kr (M.-S.L.); 2 Research Institute for Women’s Health, Sookmyung Women’s University, Seoul 04310, Korea; 3 School of Life Sciences, BK21 FOUR KNU Creative BioResearch Group, College of National Sciences, Kyungpook National University, Daegu 41566, Korea; rhr4757@knu.ac.kr; 4 Department of Obstetrics and Gynecology, Kyung Hee University Medical Center, 23, Seoul 02447, Korea; intro4med@naver.com; 5 Department of Biology Education, College of Education, Pusan National University, Busan 46241, Korea; wchang1975@pusan.ac.kr

**Keywords:** breast cancer, vasculogenic mimicry, epithelial–mesenchymal transition, AXL, miR-34a

## Abstract

Targeting the tumor vasculature is an attractive strategy for cancer treatment. However, the tumor vasculature is heterogeneous, and the mechanisms involved in the neovascularization of tumors are highly complex. Vasculogenic mimicry (VM) refers to the formation of vessel-like structures by tumor cells, which can contribute to tumor neovascularization, and is closely related to metastasis and a poor prognosis. Here, we report a novel function of AXL receptor tyrosine kinase (AXL) in the regulation of VM formation in breast cancer cells. MDA-MB-231 cells exhibited VM formation on Matrigel cultures, whereas MCF-7 cells did not. Moreover, AXL expression was positively correlated with VM formation. Pharmacological inhibition or AXL knockdown strongly suppressed VM formation in MDA-MB-231 cells, whereas the overexpression of AXL in MCF-7 cells promoted VM formation. In addition, AXL knockdown regulated epithelial–mesenchymal transition (EMT) features, increasing cell invasion and migration in MDA-MB-231 cells. Finally, the overexpression of microRNA-34a (miR-34a), which is a well-described EMT-inhibiting miRNA and targets AXL, inhibited VM formation, migration, and invasion in MDA-MB 231 cells. These results identify a miR-34a–AXL axis that is critical for the regulation of VM formation and may serve as a therapeutic target to inhibit tumor neovascularization.

## 1. Introduction

Breast cancer is the most commonly occurring cancer in women and one of the leading causes of death in women after menopause [1]. Breast cancer can be classified according to three cellular markers: (i) progesterone receptor (PR) or estrogen receptor (ER) positive, (ii) human epidermal growth factor receptor-2 (HER2) positive, and PR and/or ER positive or negative, and (iii) triple negative breast cancer (TNBC) [2,3,4]. In particular, TNBC refers to tumors without the expression of PR, ER, and HER2, and is associated with a more frequent relapse and higher mortality than the different types of breast cancer [4]. TNBC accounts for about 15–20% of all breast cancers and 5% of all cancer-related deaths [5]. Since TNBC patients cannot be treated with drugs targeting ER and HER2, they have an increased risk of low survival and disease progression [6,7]. Because current therapies for TNBC have limited effects, investigating TNBC tumor progression and development, as well as the causal agents for the phenotypic heterogeneity, is important for the development of new treatment options [3].

Vasculogenic mimicry (VM) refers to a blood supply system that is formed in the tumor cells themselves, and not by vascular endothelial cells, such as in angiogenesis. These systems appear in malignant tumors and contribute to an aggressive disease, rapid tumor growth, and a poor prognosis [8,9,10,11]. It has been identified that various factors can regulate VM, including vascular endothelial cadherin, ephrin type-A receptor 2, focal adhesion kinase, extracellular signal-regulated kinase, and matrix metalloproteinase (MMP) [11,12,13]. Interestingly, epithelial–mesenchymal transition (EMT)-related regulatory factors are upregulated in tumor cells with VM formation and are shown to play a key role in VM formation [14]. For example, Twist, a major EMT inducer, regulates the transcription of downstream genes that are responsible for VM formation [15,16].

AXL is a receptor tyrosine kinase, a component of the TAM receptors (TYRO3, AXL, and MER). In the tumor microenvironment, AXL signaling is activated through multiple mechanisms to promote tumor progression. Furthermore, AXL signaling in tumors promotes cancer stem cell-like phenotypes, drug resistance, metastasis, and EMT [17,18]. AXL expression is higher in tumor cells than in normal tissues, and it is associated with drug resistance in various cancers. AXL signaling in tumor cells is tightly controlled by various regulators, such as epigenetic and tumor microenvironment regulators. Most of the AXL signaling is triggered by its ligand, growth arrest-specific gene 6 (GAS6); however, hypoxic conditions in the tumor microenvironment also play a critical role in the activation of GAS6-AXL signaling [17,18,19,20]. While many studies show that the role of AXL is closely associated with EMT in many types of cancer, the role of AXL in VM formation has not been investigated.

MicroRNAs (miRNAs) are endogenous non-coding RNAs composed of 18–24 nucleotides, which play an essential role in biological processes by regulating gene expression [10,11]. As key regulators of gene expression, miRNAs are widely implicated in metastasis, tumorigenesis, and drug resistance [21,22]. In addition, accumulating studies provide evidence that miRNAs play an important regulatory role in the formation of VM in many types of cancer [10,23,24]. AXL expression is directly regulated by tumor suppressor miRNAs, such as miR-199a/b and miR-34a [25,26], and is closely associated with EMT in various types of cancer [27,28]. Although it was demonstrated that miR-34a directly regulates AXL expression, the role of the miR-34a–AXL axis in the formation of VM in breast cancer cells remains to be investigated.

In this study, we sought to define the role of AXL in VM formation and to understand the mechanism underlying VM formation in breast cancer cells. Here, we provide experimental evidence that the miR-34a–AXL axis is critical for the regulation of VM formation via the regulation of EMT, and it may serve as a potential therapeutic target to overcome the limitations of anti-angiogenic therapy in breast cancer.

## 2. Materials and Methods

### 2.1. Cell Culture

The human breast cancer cell lines BT-549, HS578T, MDA-MB-231, MDA-MB-468, T47D, and MCF-7 were obtained from the National Cancer Institute (NCI, USA). One human breast cancer cell line (HCC-70) was obtained from the Korean Cell Line Bank (KCLB, Seoul, Korea). MDA-MB-231 and MCF-7 human breast cancer cell lines were maintained in DMEM/HIGH GLUCOSE (HyClone, Marlborough, MA, USA) with 10% fetal bovine serum (FBS, HyClone) supplemented with 1% penicillin–streptomycin (Welgene, Daegu, Republic of Korea) and MycoZap (Lonza, Basel, Switzerland). BT-549, HS578T, MDA-MB-468, T47D, and HCC-70 human breast cancer cell lines were maintained in RPMI-1640 medium (HyClone) supplemented with 10% FBS (HyClone) supplemented with 1% penicillin–streptomycin (Welgene) and MycoZap (Lonza). All breast cancer cell lines were cultured in a 5% CO_2_ incubator at 37 °C. A stable MCF-7 cell line overexpressing AXL was produced via puromycin selection. Human umbilical vein endothelial cells (HUVECs; Lonza and Yale VBT Core, New Haven, CT, USA) were cultured in a 5% CO_2_ incubator at 37 °C with EBM-2 basal medium supplemented with EGM-2 (Lonza) with 1% penicillin–streptomycin (Welgene). HUVECs (passages 3–7) were grown to 70% to 90% confluence.

### 2.2. Transfection

Lipofectamine RNAimax (Invitrogen, Carlsbad, CA, USA) was used for siRNA and miRNA transfection, and Lipofectamine 2000 (Invitrogen) was used for the AXL lentiviral vector (abm) transfection, following the manufacturer’s instructions. AccuTarget™ AXL siRNA, negative control siRNA, miR-34a mimic, anti-miR-34a, miRNA negative control #1, and anti-miRNA negative control #1 were purchased from Bioneer. Negative control siRNA (Bioneer, Daejeon, Republic of Korea), miRNA negative control #1, and anti-miRNA negative control #1 were used as negative controls.

### 2.3. Quantitative Real-Time PCR

Total RNA was isolated using the miRNeasy RNA isolation kit (Qiagen, Hilden, Germany). Purified RNA was reverse-transcribed using the TaqMan miRNA Reverse Transcription kit (Applied Biosystems, Foster City, CA USA). miRNA quantitative real-time PCR (qRT-PCR) was performed using the TaqMan Universal Master Mix II, no UNG (Applied Biosystems), and miR-34a was detected using Taqman probes. RNU6B was used as an internal control for the normalization of miR-34a. mRNA was reverse-transcribed using the qPCRBIO cDNA Synthesis kit (PCR Biosystems, London, UK). qPCR was performed using qPCRBIO syGreen Mix Lo-ROX (PCR Biosystems) according to the manufacturer’s instructions. The internal control used was ribosomal 18s RNA. The sequences of the PCR primers are listed in Table 1.

### 2.4. Western Blotting

Cells were lysed using RIPA buffer (Biosesang, Seongnam, Republic of Korea) containing protease inhibitor cocktails (Gendepot). Subsequently, cell lysates were centrifuged at 13,000 rpm at 4 °C for 15 min. Protein quantification was performed using the protein assay dye reagent (Bio-Rad, Hercules, CA, USA), and equal protein concentrations were boiled, loaded into sodium dodecyl sulfate-polyacrylamide gels, and transferred to a polyvinyl difluoride membrane (Millipore, Burlington, MA, USA). Protein bands were hybridized with the primary antibodies against AXL (1:1000, Cell Signaling, Danvers, MA, USA), E-cadherin (1:1000, Cell Signaling), N-cadherin (1:1000, BD, Franklin Lakes, NJ, USA), vimentin (1:3000, Santa Cruz, Dallas, TX, USA), Slug (1:1000, Cell Signaling), and GAPDH (1:5000, Cell Signaling). Immunodetection was conducted using HRP-conjugated secondary antibodies, and development was performed using an enhanced chemiluminescent detection system (Thermo Scientific, Waltham, MA, USA).

### 2.5. Cell Migration Assay

After seeding MDA-MB-231 breast cancer cells in a 12-well plate and growing them to 70–80% confluence, siRNA or miRNA mimics were transfected using lipofectamine RNAimax (Invitrogen, Carlsbad, CA, USA). After 48 h, transfected MDA-MB-231 breast cancer cells were cultured overnight in complete media diluted with 20 mM thymidine. The next day, the center of the well was scratched using a p200 pipette tip. The cells were incubated in complete media containing thymidine. The width of the gaps was calculated using ImageJ software (version 1.53a).

### 2.6. Three-Dimensional Culture Assay

Well plates (48 wells) were coated with Matrigel Matrix (Growth Factor Reduced, Phenol-free, BD Bioscience, Franklin Lakes, NJ, USA) and hardened at 37 °C. Then, breast cancer cells, siRNA or miRNA mimic-transfected MDA-MB-231 breast cancer cells, AXL-overexpressing MCF-7 breast cancer cells, or R428 (MedChemExpress Life Science Reagents, Monmouth Junction, NJ, USA)-treated MDA-MB-231 breast cancer cells were cultured in serum-free media on Matrigel-coated plates. For co-culture three-dimensional culture assays, MDA-MB-231 breast cancer cells and HUVECs were fluorescent-labelled with 1 μM Cell Tracker Orange or Cell Tracker Green (Thermo Fisher Scientific, Waltham, MA, USA), respectively, for 30 min at 37 °C in serum-free media. Mixed cells (0.5 × 10^4^ MDA-MB-231 breast cancer cells +1 × 10^4^ HUVECs per well) were then seeded on Matrigel. Analysis of the formed tube length was performed using ImageJ software (version 1.53a).

### 2.7. Transwell Invasion Assay

Transwell chambers were coated with Matrigel diluted with serum-free media. For the invasion assay, cells were seeded with serum-free media into the upper chamber, and complete media was added to the bottom chamber. After 24–48 h, the medium in the upper chamber was removed and washed with 1× PBS. The cells were fixed in 3.7% formaldehyde. The permeabilization step was performed using 100% methanol, and then cells were stained with 0.5% crystal violet diluted in 20% methanol. Cell counting was performed using ImageJ software (version 1.53a).

### 2.8. Statistical Analysis

All experiments were performed at least three times and analyzed using GraphPad Prism 5.0 software. Unpaired two-tailed Student’s t-test was performed to assess the statistical differences. A *p* < 0.05 was considered statistically significant (* *p* < 0.05; ** *p* < 0.01; *** *p* < 0.001).

## 3. Results

### 3.1. Expression Levels of AXL are Associated with Vasculogenic Mimicry Formation In Vitro

**Figure 1:**
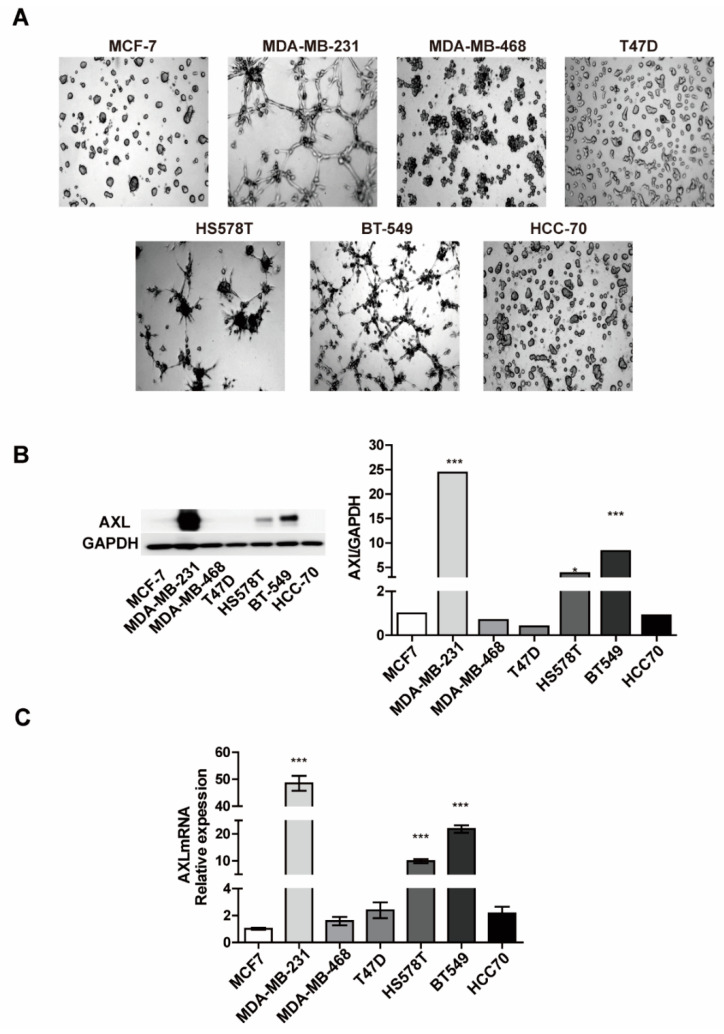
AXL expression is involved in vasculogenic mimicry formation in breast cancer cell lines. (**A**) Vasculogenic mimicry (VM) formation ability in breast cancer cell lines. (**B**) AXL protein expression in breast cancer cell lines. (**C**) AXL mRNA expression in breast cancer cell lines. * *p* < 0.05, *** *p* < 0.001 versus controls, calculated using unpaired two-tailed Student’s *t*-test. Error bars, S.E.M.

To investigate the involvement of AXL in VM formation in breast cancer cells, VM formation and AXL expression were examined in seven breast cancer cell lines. We conducted a three-dimensional culture assay of MDA-MB-231, MDA-MB-468, T47D, HS578T, BT-549, HCC-70, and MCF-7 breast cancer cell lines. As shown in Figure 1A, Supplementary Materials Videos S1 and S2, the differential abilities for VM formation were observed among the seven breast cancer cell lines. MDA-MB-231 breast cancer cells had similar abilities of VM formation as endothelial cells, whereas BT-549 and HS578T breast cancer cells exhibited a relatively lower VM formation ability. However, no VM formation was observed in the other breast cancer cell lines. Next, we evaluated the expression level of AXL in seven breast cancer cell lines to investigate the correlation with VM formation. Both the protein and mRNA expression levels of AXL in MDA-MB-231 breast cancer cells were substantially higher than those in the other breast cancer cell lines (Figure 1B,C). These data indicate that breast cancer cells with a higher AXL expression were more likely to form VM, compared to cells with low AXL expression levels, thereby suggesting that the expression levels of AXL are associated with the VM formation potential in breast cancer cells.

### 3.2. Inhibition of AXL Suppresses Vasculogenic Mimicry Formation in MDA-MB-231 Breast Cancer Cells

**Figure 2:**
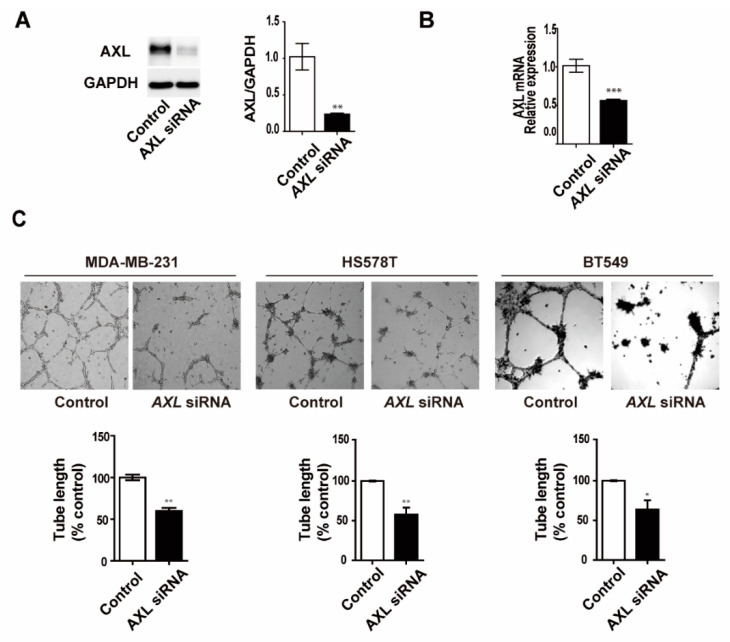

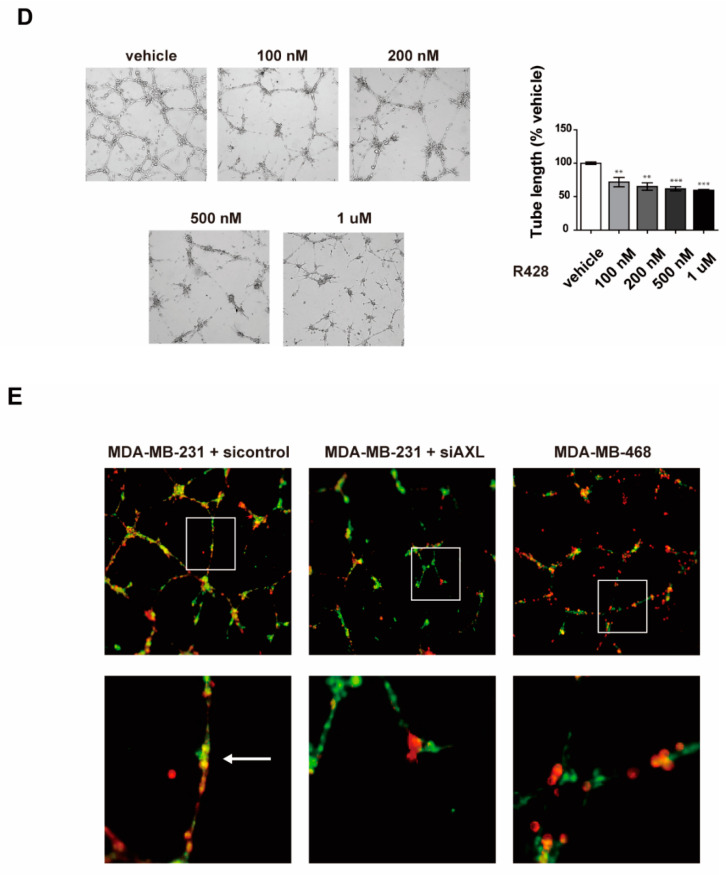
Inhibition of AXL suppressed vasculogenic mimicry and mosaic cords formation in MDA-MB-231 breast cancer cells. (**A**) AXL protein and (**B**) mRNA expression in AXL-knockdown MDA-MB-231 breast cancer cells. (**C**) Inhibition of VM formation in response to AXL knockdown by siRNA in MDA-MB-231, BT-549, and HS578T breast cancer cells. (**D**) Inhibition of VM formation via R428 treatment at 100 nM, 200 nM, 500 nM, and 1 µM in MDA-MB-231 breast cancer cells. (**E**) Co-culture three-dimensional culture assays with breast cancer cells (red) and HUVECs (green). Mosaic cord structure (arrow). * *p* < 0.05, ** *p* < 0.01, *** *p* < 0.001 versus controls, calculated using unpaired two-tailed Student’s *t*-test. Error bars, S.E.M.

To assess the effect of AXL inhibition on VM formation in MDA-MB-231 breast cancer cells, we first assessed the siRNA knockdown effect by examining the protein and mRNA expression levels of AXL. The protein and mRNA expression of AXL was significantly decreased in the knockdown cells compared to the control cells (Figure 2A,B). Next, we determined whether the knockdown of AXL can affect the formation of VM in AXL high-expressing breast cancer cells. AXL knockdown led to a drastic reduction in VM formation in MDA-MB-231, BT-549, and HS578T breast cancer cells, suggesting the potential role of AXL as a mediator of VM formation (Figure 2C). To further investigate the role of AXL in VM formation, MDA-MB-231 breast cancer cells were treated with varying concentrations of R428, an AXL inhibitor, and its effects on VM formation were observed. All concentrations of R428 significantly inhibited VM formation (Figure 2D). Altogether, AXL inhibition was sufficient to abolish VM formation ability, suggesting the important role of AXL in this process.

Previous studies showed that tumor cells form mosaic vessels with endothelial cells [29,30,31], providing a blood supply to tumor cells. Therefore, we next investigated cooperative interaction between breast cancer cells and HUVECs in co-cultures on Matrigel. We found that a mosaic cords structure was observed in the mixture of AXL high-expressing MDA-MB-231 cells and HUVECs on Matrigel, whereas AXL low-expressing MDA-MB-468 cells did not form mosaic cords with HUVECs. These findings suggest that the mosaic cords may serve as a bridge for the blood flow. Furthermore, AXL silencing MDA-MB-231 cells exhibited a relatively lower mosaic cords formation with HUVECs compared to control MDA-MB-231 cells, further demonstrating the role of AXL in mosaic cords formation (Figure 2E).

### 3.3. Inhibition of AXL Suppresses Cell Invasion and Migration in MDA-MB-231 Breast Cancer Cells

**Figure 3:**
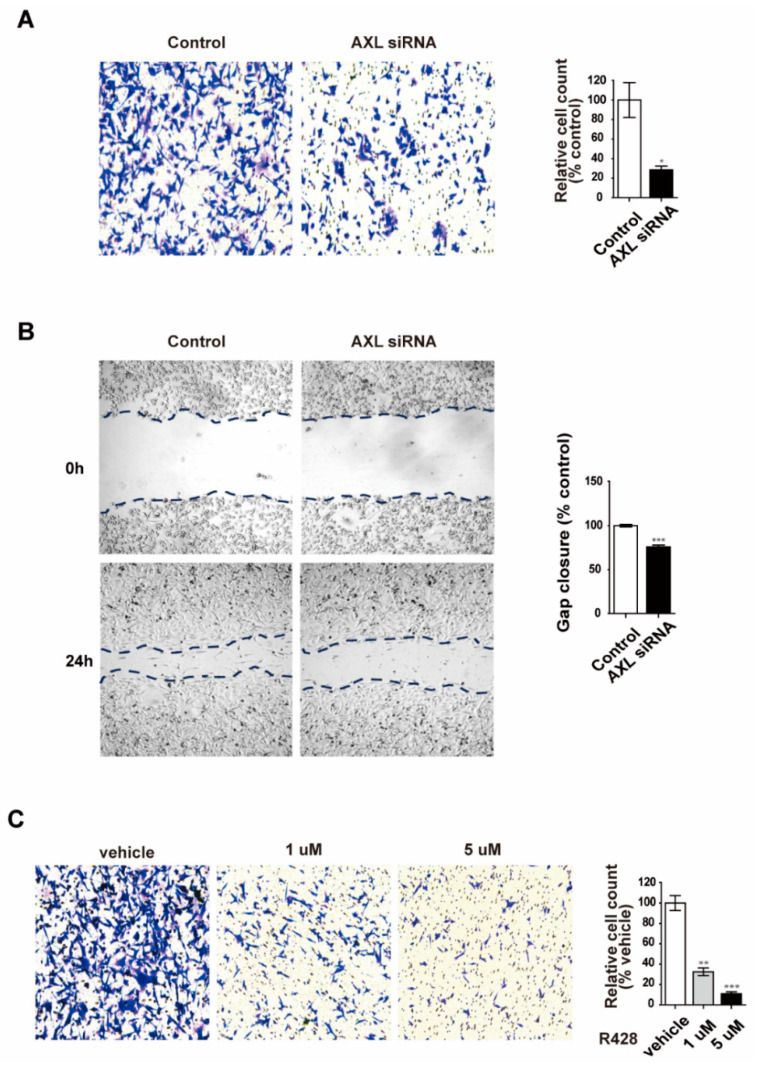
Inhibition of AXL suppresses cell invasion and migration in MDA-MB-231 breast cancer cells. (**A**) Inhibition of invasion ability in MDA-MB-231 breast cancer cells after AXL knockdown. (**B**) MDA-MB-231 breast cancer cell migration inhibition following AXL knockdown. (**C**) Inhibition of invasive ability in R428-treated MDA-MB-231 breast cancer cells. * *p* < 0.05, ** *p* < 0.01, *** *p* < 0.001 versus controls, calculated using unpaired two-tailed Student’s *t*-test. Error bars, S.E.M.

Many studies have shown that VM is highly associated with aggressive tumor features and shorter overall survival [32,33,34]. Additionally, AXL has been implicated in metastasis, invasion, and migration in diverse human cancers [28,35]. Basing our research on these studies, we set out to investigate the role of AXL in invasion and migration of MDA-MB-231 breast cancer cells. We found that AXL knockdown caused a significant decrease in the invasion and migration of MDA-MB-231 breast cancer cells (Figure 3A,B). In addition, AXL inhibition by R428 decreased the motility of MDA-MB-231 cells (Figure 3C). These results suggest AXL is associated with invasiveness and VM ability of MDA-MB-231 cells.

### 3.4. Overexpression of AXL Promotes Vasculogenic Mimicry Formation and Regulates Expression of Epithelial–Mesenchymal Transition Markers in MCF-7 Breast Cancer Cells

**Figure 4:**
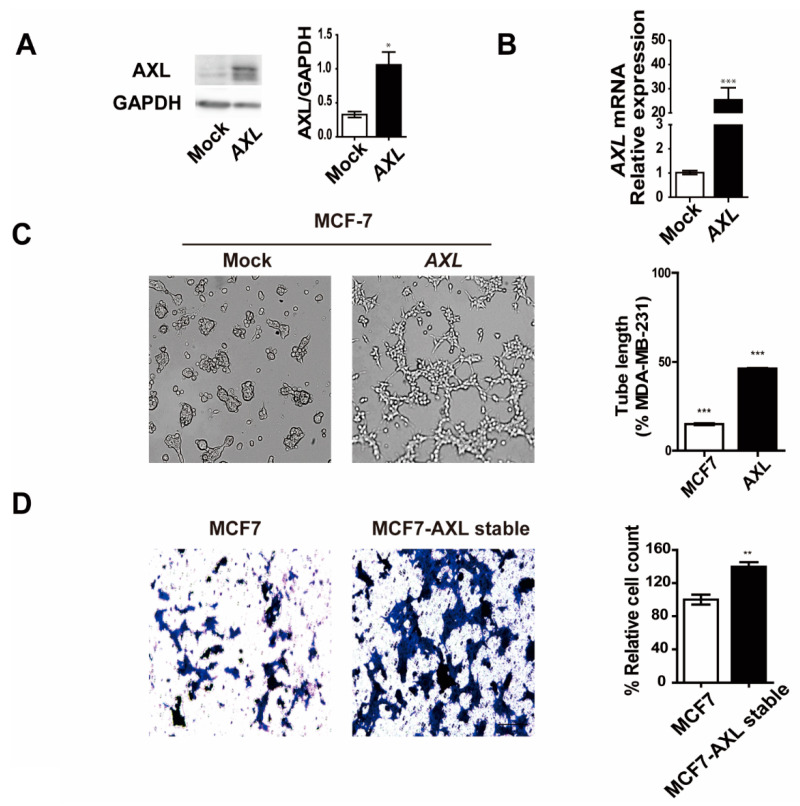

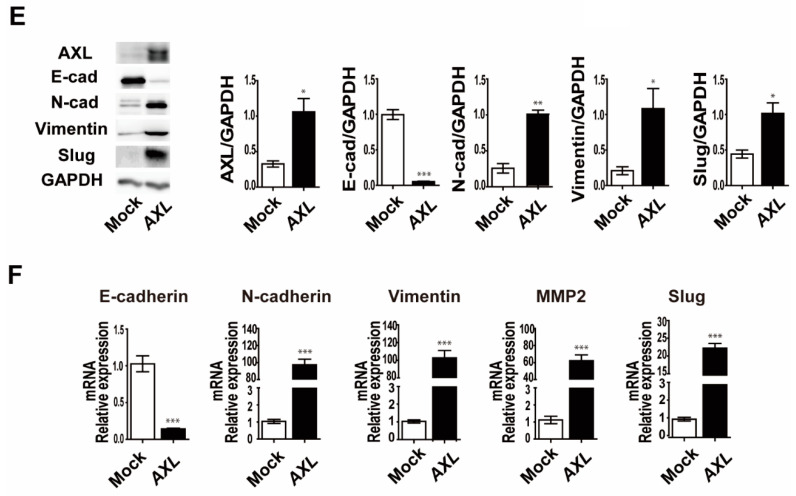
Overexpression of AXL induces vasculogenic mimicry formation and regulates epithelial–mesenchymal transition (EMT) marker expression in MCF-7 breast cancer cells. (**A**) AXL protein and (**B**) mRNA expression in MCF-7 breast cancer cells and MCF-7/AXL stable cells. (**C**) Development of VM due to AXL overexpression in MCF-7 breast cancer cells. (**D**) Enhanced invasion ability in MCF-7/AXL stable cells. (**E**) EMT marker protein and (**F**) mRNA expression in MCF-7/AXL stable cells. * *p* < 0.05, ** *p* < 0.01, *** *p* < 0.001 versus controls, calculated using unpaired two-tailed Student’s *t*-test. Error bars, S.E.M.

To further characterize AXL-mediated VM formation, MCF-7 breast cancer cells were transfected with a lentiviral vector expressing AXL. Stable clones were isolated using puromycin and analyzed for AXL expression in the AXL-overexpressing MCF-7 (MCF-7/AXL) stable cell line. The expression levels of AXL protein and mRNA significantly increased in the MCF-7/AXL stable cells (Figure 4A,B). Next, VM formation was observed using a three-dimensional culture assay. MCF-7 control cells did not form a tube, but the MCF-7/AXL stable cells exhibited VM formation (Figure 4C). Furthermore, MCF-7/AXL stable cells showed enhanced invasion ability (Figure 4D). These results further demonstrate the role of AXL in VM formation and invasiveness of breast cancer cells. Next, to determine the mechanism of AXL in VM formation and invasiveness of breast cancer cells, we examined the expression of EMT markers in MCF-7/AXL stable cells. Consequently, we found that the expression of E-cadherin, which is an epithelial marker, decreased, and the expressions of N-cadherin, vimentin, Slug, and MMP2, which are mesenchymal markers, increased (Figure 4E,F). Collectively, these results suggest that AXL regulates the expression of EMT markers in MCF-7 cells and may provide evidence of an association between VM formation and invasiveness of breast cancer cells.

### 3.5. miR-34a Regulates AXL Expression and Reduces Cell Invasion, Migration, and Vasculogenic Mimicry Formation in MDA-MB-231 Breast Cancer Cells

**Figure 5:**
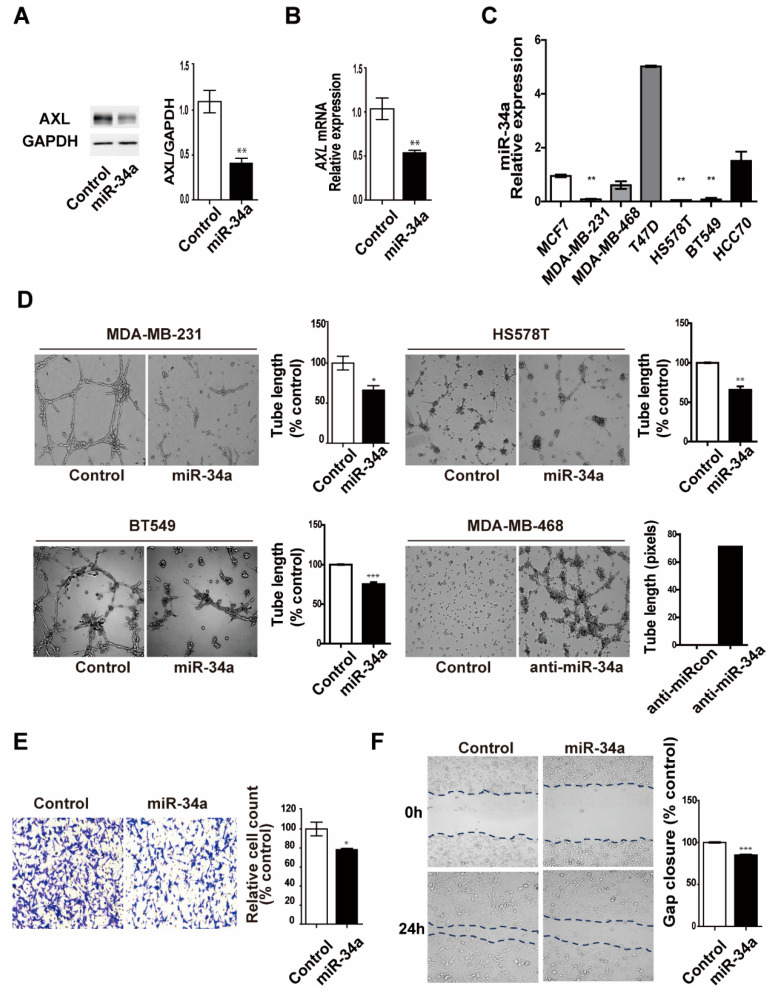
AXL-targeting miR-34a regulates cell invasion, migration, and vasculogenic mimicry formation in MDA-MB-231 breast cancer cells. (**A**) AXL protein and (**B**) mRNA expression in response to overexpression of miR-34a mimics or inhibition of miR-34a with anti-miRs in MDA-MB-231 breast cancer cells. (**C**) miR-34a expression in breast cancer cell lines. (**D**) VM formation ability in response to overexpression of miR-34a or inhibition of miR-34a with anti-miRs in MDA-MB-231 and MDA-MB-468 breast cancer cells, respectively. (**E**) Invasion and (**F**) migration by overexpression of miR-34a mimics in MDA-MB-231 breast cancer cells. * *p* < 0.05, ** *p* < 0.01, *** *p* < 0.001 versus controls, calculated using unpaired two-tailed Student’s *t*-test. Error bars, S.E.M.

In previous studies, miR-34a was shown to be a tumor suppressor that acts by targeting multiple genes in various cancers. AXL is overexpressed in various cancers and promotes tumor metastasis and tumorigenesis, which is also known to be inhibited by miR-34a [36,37]. We confirmed that the overexpression of miR-34a in MDA-MB-231 breast cancer cells significantly decreased AXL expression at both the protein and mRNA levels (Figure 5A,B). In addition, we also found a significant inverse correlation between the levels of miR-34a and those of AXL in seven breast cancer cell lines (Figure 5C). As assessed using qRT-PCR, miR-34a expression was markedly lower in MDA-MB-231, HS578T, and BT-549 breast cancer cells, which exhibited a higher VM formation ability compared with the other breast cancer cell lines. These results suggest that AXL is regulated by miR-34a, which may play a key role in the regulation of VM formation in breast cancer cells. To investigate the relationship between miR-34a and AXL functions in VM formation and invasiveness of breast cancer cells, we determined the effects of miR-34a overexpression on invasion, migration, and VM formation in AXL high-expressing breast cancer cells. As shown in Figure 5D, we demonstrated that miR-34a overexpression significantly inhibits VM formation in MDA-MB-231, BT-549, and HS578T breast cancer cells, whereas inhibition of miR-34a exhibited VM formation of MDA-MB-468 breast cancer cells. Finally, we demonstrated that miR-34a overexpression inhibited invasion and cell migration in MDA-MB-231 cells (Figure 5E,F). Taken together, these results suggest that miR-34a regulates invasiveness and VM formation via regulation of AXL expression in breast cancer cells.

## 4. Discussion

Given that angiogenesis is an indispensable process for tumor growth and metastasis, targeting angiogenesis is considered as a cancer treatment strategy, but certain limitations still remain [38]. Tumor vasculature is strikingly heterogeneous and fundamentally different from the normal vasculature. In addition, many studies provide evidence that VM serves as a key alternative process for tumor neovascularization under an insufficient blood supply of oxygen and nutrients to the tumor tissue. VM is an alternative tumor vascularization mechanism that is directly built inside tumor cells to provide them with nutrients, which differs from angiogenesis via activation of the pre-existing host endothelium [10,11,39]. Thus, VM targeting may represent a promising therapeutic strategy and overcome the limitations of anti-angiogenic treatment of cancer patients. In this study, we identified an miRNA-driven regulation of AXL expression and demonstrated its involvement in the regulation of AXL-mediated VM formation and invasiveness of breast cancer cells.

We obtained several important conclusions from the current study. First, AXL expression correlates with VM formation in vitro. Second, inhibition of AXL suppresses cell invasion and migration in MDA-MB-231 cells. Third, AXL inhibition suppresses VM and mosaic cords formation in MDA-MB-231 cells. Fourth, AXL overexpression regulates the expression of EMT markers, while promoting VM formation in MCF-7 cells. Lastly, miR-34a inhibits AXL expression and regulates VM formation, cell invasion, and migration in MDA-MB-231 breast cancer cells.

In various cancers, AXL is known to regulate the proliferation, survival, angiogenesis, invasion, and migration of tumor cells. In particular, a high expression of AXL in malignant tumors has been associated with a poor prognosis [40]. AXL is overexpressed in mesenchymal EMT-like cells, promoting resistance to targeted chemotherapy [41,42]. Moreover, AXL mediates immune evasion and drug resistance [43] and significantly promotes angiogenesis [44]. The role of AXL in cancer progression and angiogenesis is well known, but the role of AXL in VM formation has not been elucidated so far. Here, we provide a novel function of AXL in VM formation in breast cancer cells. Breast cancer cells with a high AXL expression formed VM, but those with a low AXL expression did not. In addition, VM formation was suppressed by AXL inhibition in MDA-MB-231, BT-549, and HS578T breast cancer cells with the highest AXL expression. Moreover, AXL overexpression in MCF-7 breast cancer cells with the lowest AXL expression promoted VM formation. These findings provide evidence, for the first time, that AXL expression levels are closely associated with VM formation in vitro, and that VM formation can be regulated via modulation of AXL expression in breast cancer cells.

To elucidate the mechanism involved in AXL-mediated VM, we investigated the possible involvement of VEGF and FGF signaling previously reported to be important in VM, invasiveness, and angiogenesis of various cancers [45,46]. However, AXL silencing did not have any effect on the FGF2, VEGF-A, FGFR1, VEGFR1, and VEGFR2 expression of MDA-MB-231 breast cancer cells (data not shown), suggesting that AXL-mediated VM formation may be independent of the VEGF/VEGFR and FGF/FGFR axis. Further studies to investigate the mechanism involved in AXL-mediated VM are needed to fully elucidate the role of AXL in breast cancer.

miRNAs are associated with the initiation, progression, and prognosis of cancer. Depending on the functions of miRNA targets, miRNAs may act as tumor suppressors or oncogenes and can be used as novel therapeutic agents for cancer treatment. Furthermore, emerging studies have shown that miRNAs play an important role in the regulation of VM formation in many cancers [10,23,24]. miR-34a has been shown to act as a tumor suppressor by regulating processes that are associated with cell cycle, proliferation, metastasis, and invasion [47,48]. In addition, a high expression of miR-34a has been closely associated with a better survival in patients with breast cancer, indicating that miR-34a is an important prognostic marker for breast cancer patients [49]. miR-34a regulates processes that are essential for tumorigenesis by targeting AXL in malignant tumors [25,26]. However, it remains to be investigated whether miR-34a-mediated regulatory mechanisms are involved in VM formation by targeting AXL in breast cancer cells.

Hence, we investigated the role of the miR-34a–AXL axis on VM formation and invasiveness in breast cancer cells because the VM is closely associated with aggressive tumor features [32,33,34]. When miR-34a was overexpressed in MDA-MB-231 breast cancer cells with a high AXL expression, cell migration and invasion, which are essential for cancer metastasis, were inhibited. More importantly, VM formation was suppressed. In addition, in MCF-7/AXL stable cells, the expression of epithelial markers was decreased, and the expression of mesenchymal markers was increased showing an increased VM formation. Together, our findings support the hypothesis that miR-34a-mediated AXL targeting affects VM formation and aggressive tumor features, and provides a novel molecular mechanism for VM formation in breast cancer cells. Given these findings, future studies will be necessary to overcome the limitations of anti-angiogenic therapy by modulating the miR-34a–AXL axis in in vivo breast cancer models.

## 5. Conclusions

In conclusion, we show that the modulation of AXL expression via miR-34a can manipulate VM formation and aggressiveness in breast cancer cells. These findings provide a basis for overcoming the limitations of existing cancer therapies and developing new therapies.

## Figures and Tables

**Table 1:** Primer sequences for targeted genes.

Name	Primers
Human 18s rRNA	Forward: 5′-ACCCGTTGAACCCCATTCGTGA-3′
	Reverse: 5′-GCCTCACTAAACCATCCAATCGG-3′
Human AXL	Forward: 5′-AGGCTGAAGAAAGTCCCTTCG-3′
	Reverse: 5′-CCCGGGCACCTGTGATATTC-3′
Human E-cadherin	Forward: 5′-GGACCTGGCAAGATGCAGAA-3′
	Reverse: 5′-GCTGCTTGGCCTCAAAATCC-3′
Human N-cadherin	Forward: 5′-CCTCCAGAGTTTACTGCCATGAC-3′
	Reverse: 5′-GTAGGATCTCCGCCACTGATTC-3′
Human Vimentin	Forward: 5′-AGGCAAAGCAGGAGTCCACTGA-3′
	Reverse: 5′-ATCTGGCGTTCCAGGGACTCAT-3′
Human MMP2	Forward: 5′-AGCGAGTGGATGCCGCCTTTAA-3′
	Reverse: 5′-CATTCCAGGCATCTGCGATGAG-3′
Human Slug	Forward: 5′-ATCTGCGGCAAGGCGTTTTCCA-3′
	Reverse: 5′-GAGCCCTCAGATTTGACCTGTC-3′

## Supplementary Materials

The following are available online at https://www.mdpi.com/2073-4425/12/1/9/s1. Video S1: VM formation ability of MDA-MB-231 breast cancer cells. Video S2: VM formation ability of MCF-7 breast cancer cells.
